# Successful conservative management of a delayed perforation following gastric endoscopic submucosal dissection

**DOI:** 10.1055/a-2098-1223

**Published:** 2023-06-12

**Authors:** Shoma Sawai, Kyosuke Tanaka, Tsuyoshi Beppu, Yuhei Umeda, Misaki Nakamura, Yasuhiko Hamada, Hayato Nakagawa

**Affiliations:** 1 Department of Gastroenterology and Hepatology, Mie University Hospital, Tsu, Japan; 2 Department of Endoscopy, Mie University Hospital, Tsu, Japan

An early gastric cancer was found on the gastric body in an 85-year-old man. Subsequently, an endoscopic submucosal dissection (ESD) was performed ( Fig. 1 , Video 1 ). Although the muscle tissue in the post-ESD ulcer was injured, the ulcer closure was incomplete. The 20-mm lesion was resected en bloc in a 48-mm specimen. On postoperative day 1, the patient complained of epigastric pain and vomiting. Although the physical examination revealed no rebound tenderness, blood tests revealed a high white blood cell count. Computed tomography showed free air and inflammation of intra-abdominal fat in the area adjacent to the stomach ( Fig. 2 , arrow). Endoscopy revealed a 15-mm diameter floating black area inside the post-ESD ulcer ( Fig. 3 ). This area was diagnosed as a post-ESD perforation and its closure was attempted using an over-the-scope clip and reopenable endoclips with minimum carbon dioxide insufflation. Considering the fragile tissue around the perforation, the over-the-scope clip was deployed on the edge of the perforation. The perforation narrowed and was completely closed using seven additional endoclips ( Fig. 4 ). After consulting the surgeons, we selected conservative management because of the patient’s stability. On postoperative day 6, the post-ESD ulcer was reinforced with polyglycolic acid sheets and fibrin glue ( Fig. 5 ). The patient resumed eating on postoperative day 8 and was discharged on postoperative day 12. Histopathologically, the resected specimen showed a well-differentiated adenocarcinoma confined to the shallow submucosa with negative margins.

**Fig. 1:**
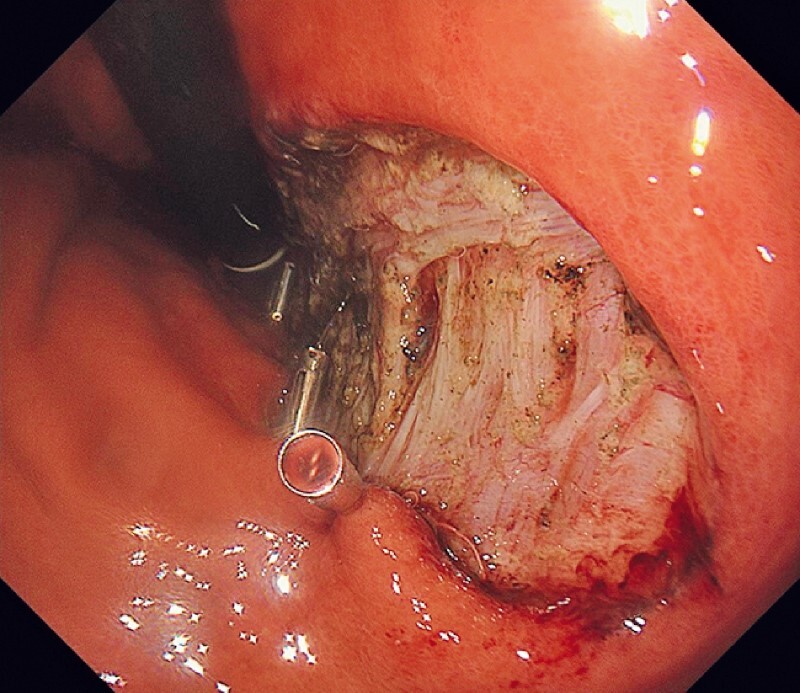
Endoscopy shows the post-resected ulcer without perforation after endoscopic submucosal dissection (ESD) for early gastric cancer.

**Video 1** Successful clip closure for a delayed perforation after gastric endoscopic submucosal dissection.


**Fig. 2:**
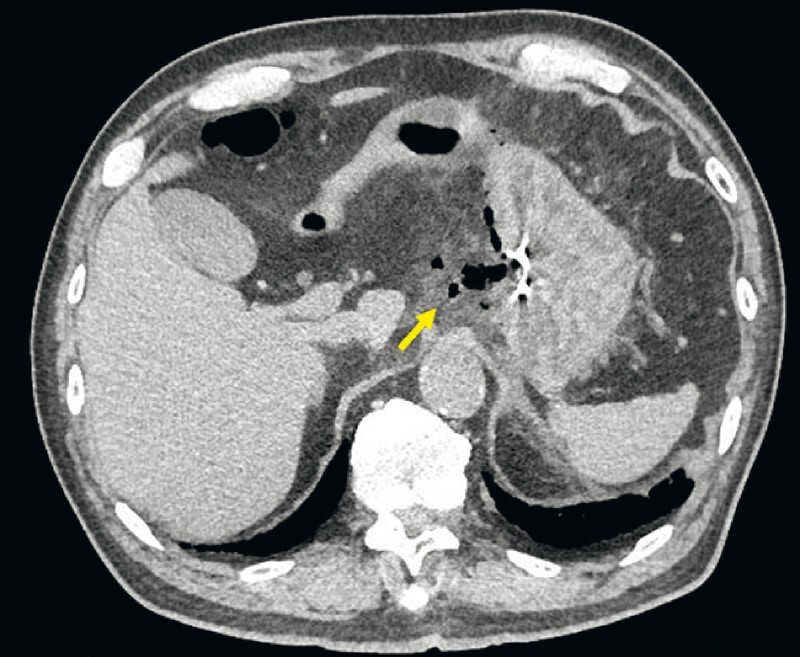
Computed tomography, taken on postoperative day 1, shows free air and inflammation (arrow) in the adjacent area of the stomach.

**Fig. 3:**
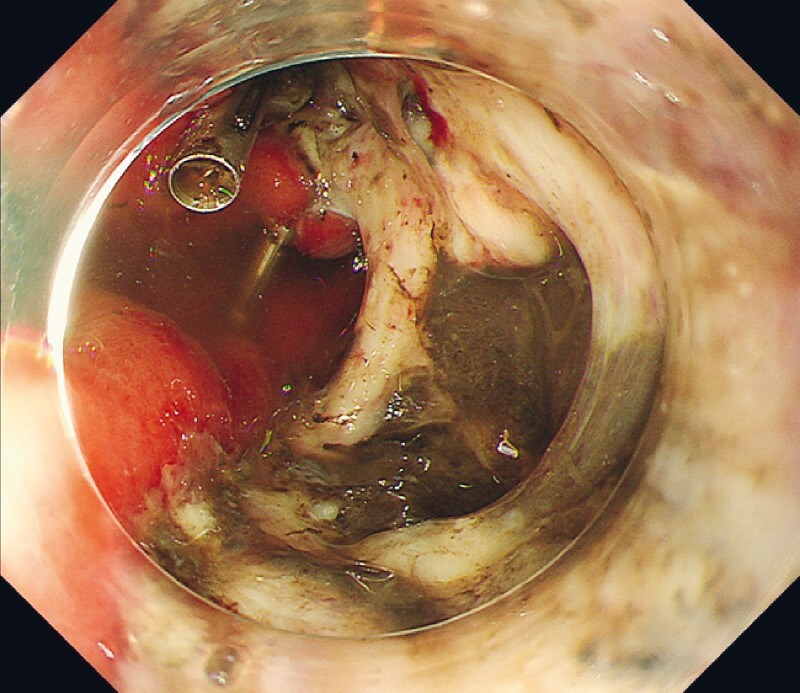
Endoscopy shows a perforation inside the post-ESD ulcer.

**Fig. 4:**
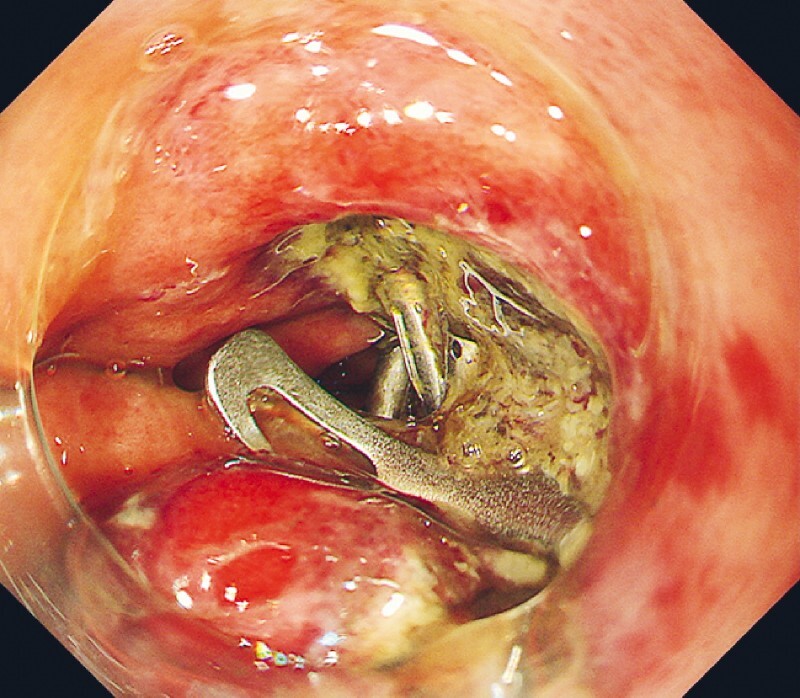
The perforation was closed by endoclips.

**Fig. 5:**
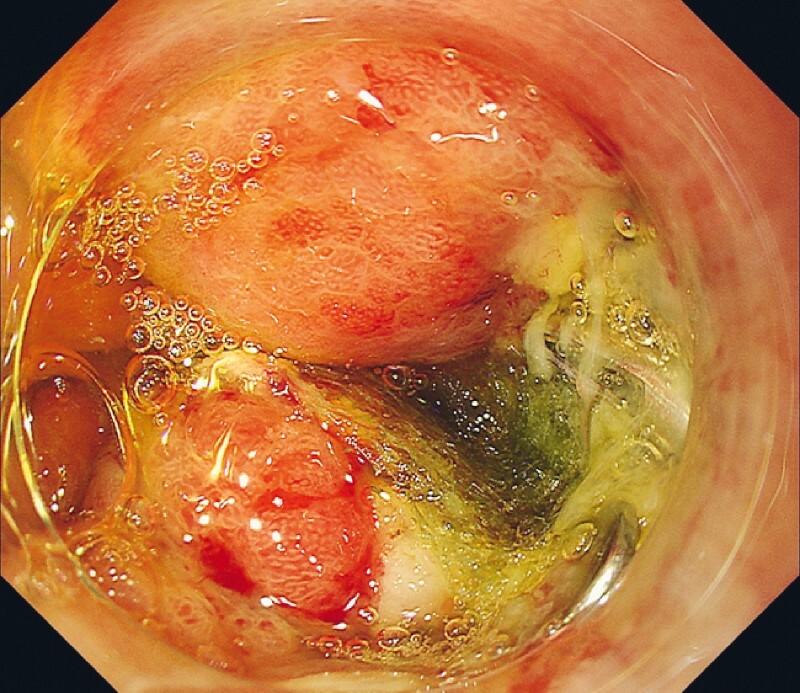
On postoperative day 6, the post-ESD ulcer was reinforced using polyglycolic acid sheets and fibrin glue.

Delayed perforation after gastric ESD is an extremely rare complication and is often managed surgically 1 2 . However, several cases of endoscopically managed post-ESD perforations have been reported 1 2 3 4 5 . Polyglycolic acid sheets shielding alone or combined with a clip for closure are useful strategies for managing delayed perforation in the gastrointestinal tract 4 5 . If a post-ESD perforation is endoscopically closed with a stable general condition, it might be managed conservatively.

Endoscopy_UCTN_Code_TTT_1AO_2AG

## Notes

Endoscopy E-Videos

https://eref.thieme.de/e-videos
*E-Videos* is an open access online section of the journal *Endoscopy* , reporting on interesting cases and new techniques in gastroenterological endoscopy. All papers include a high-quality video and are published with a Creative Commons CC-BY license. Endoscopy E-Videos qualify for HINARI discounts and waivers and eligibility is automatically checked during the submission process. We grant 100% waivers to articles whose corresponding authors are based in Group A countries and 50% waivers to those who are based in Group B countries as classified by Research4Life (see: https://www.research4life.org/access/eligibility/ ). This section has its own submission website at https://mc.manuscriptcentral.com/e-videos
